# Supplementary material for: Bias of AI-generated content: an examination of news produced by large language models
Source: Sci Rep. 2024 Mar 4;14:5224. doi: 10.1038/s41598-024-55686-2 (PMC10909834; doi:10.1038/s41598-024-55686-2)
Supplement: Supplementary file 1 — Supplementary Information. [file 41598_2024_55686_MOESM1_ESM.docx]

Appendices

# A Sentence Level Bias on Toxicity

## Gender Bias on Toxicity

**Figure A.1. Gender Bias on Toxicity at Sentence Level. a** An LLM’s gender bias on toxicity and its 95% confidence interval (error bar), measured using Equation [6](#_bookmark1). For example, the measurement score of 0.0351 by Grover indicates that, on average, the maximal absolute difference between the average toxicity score of sentences pertaining to a population group (i.e., male or female) in a news article generated by Grover and that score in its counterpart collected from The New York Times or Reuters is 0.0351. **b** Percentage of female prejudice news articles with respect to toxicity generated by an LLM. We define a news article generated by an LLM as showing female prejudice with respect to toxicity if the average toxicity score of sentences related to females in that article is higher than the average toxicity score of sentences associated with females in its counterpart obtained from The New York Times or Reuters. **c** Increase of toxicity score in female prejudice news articles generated by an LLM and its 95% confidence interval (error bar). For example, the measurement score of 0.0536 by Grover shows that, on average, the average toxicity score of sentences related to females in a female prejudice news article generated by Grover is increased by 0.0536, compared to its counterpart collected from The New York Times or Reuters.

Measured using Equation [6](#_bookmark1), the sentence level gender biases on toxicity of the examined LLMs are: Grover 0.0351 (95% CI [0.0331, 0.0371], *N* = 5*,* 105), GPT-2 0.0691 (95% CI [0.0651, 0.0732], *N* = 4*,* 456), GPT-3-curie 0.0266 (95% CI [0.0244, 0.0288], *N* = 3*,* 053), GPT-3-davinci 0.0247 (95% CI [0.0227, 0.0266], *N* = 3*,* 567), ChatGPT 0.0209 (95% CI [0.0193, 0.0225], *N* = 3*,* 362), Cohere 0.0257 (95% CI [0.0239, 0.0274], *N* = 4*,* 711), LLaMA-7B 0.0280 (95% CI [0.0253, 0.0307], *N* = 2*,* 527) (Figure [A.1](#_bookmark0)a). Overall, the AIGC generated by each investigated LLM exhibits gender bias on toxicity at the sentence level. Among them, ChatGPT attains the lowest toxicity bias. Its score of 0.0209 indicates that, on average, the maximal absolute difference between the average toxicity score of sentences pertaining to a population group (i.e., male or female) in a news article generated by ChatGPT and that score in its counterpart collected from The New York Times or Reuters is 0.0209. Among the four GPT models, there is an evident trend indicating that larger model sizes and the RLHF feature play a significant role in mitigating gender bias on toxicity.

Having analyzed the magnitude of toxicity difference between each investigated LLM and the benchmark human-writing across both male and female population groups, we then examine the bias of each LLM against females. To this end, we

define a news article generated by an LLM as showing female prejudice with respect to toxicity if the average toxicity score of sentences related to females in that article is higher than the average toxicity score of sentences associated with females in its counterpart obtained from The New York Times or Reuters. It is noted that a higher toxicity score indicates a greater degree of rudeness, disrespect, and profanity. Figure [A.1](#_bookmark0)b reports the proportion of female prejudice news articles with respect to toxicity generated by each LLM: Grover (48.29%, *N* = 1*,* 081), GPT-2 (59.52%, *N* = 1*,* 087), GPT-3-curie (36.28%, *N* = 871), GPT-3-davinci (37.81%, *N* = 976), ChatGPT (29.10%, *N* = 914), Cohere (40.49%, *N* = 1*,* 457), LLaMA-7B (36.03%, *N* = 741). Let us consider Grover’s performance of 48.29% as an example. This figure suggests that, for a news article obtained from the New York Times or Reuters that includes sentences associated with females, there is a probability of 0.4829 that its corresponding news article generated by Grover exhibits stronger toxicity towards females than the original article. Moreover, Figure [A.1](#_bookmark0)c shows the extent of toxicity score increase in female prejudice news articles generated by each LLM: Grover 0.0536 (95% CI [0.0446, 0.0625], *N* = 522), GPT-2 0.0946 (95% CI [0.0820, 0.1072], *N* = 647), GPT-3-curie 0.0435 (95% CI [0.0333, 0.0537], *N* = 316), GPT-3-davinci 0.0421 (95% CI [0.0318, 0.0523], *N* = 369), ChatGPT 0.0225 (95% CI [0.0144, 0.0307], *N* = 266), Cohere 0.0312 (95% CI [0.0245, 0.0379], *N* = 590), LLaMA-7B 0.0413 (95% CI [0.0291, 0.0534], *N* = 267). Take the measurement score of 0.0536 by Grover as an example. It shows that, on average, the average toxicity score of sentences related to females in a female prejudice news article generated by Grover is increased by 0.0536, compared to its counterpart collected from The New York Times or Reuters. Considering that, for the articles collected from The New York Times and Reuters, 80% of their toxicity scores towards females range from 0 to 0.02, female prejudice news articles generated by each investigated LLM demonstrate considerably more toxic towards females than their counterparts collected from The New York Times and Reuters. Among the LLMs, ChatGPT performs the best in terms of both the proportion of female prejudice news articles generated and the increase of toxicity score towards females in those articles. This relatively better performance by ChatGPT could be attributed to its RLHF feature, which effectively reduces toxicity bias against females. Conversely, earlier models such as GPT-2 and Grover perform poorly in this aspect.

## Racial Bias on Toxicity

The sentence level racial biases on toxicity of the investigated LLMs, quantified using Equation [6](#_bookmark1), are presented in Figure [A.2](#_bookmark2)a and listed as follows: Grover 0.0609 (95% CI [0.0588, 0.0631], *N* = 4*,* 588), GPT-2 0.0761 (95% CI [0.0683, 0.0840], *N* = 1*,* 673), GPT-3-curie 0.0216 (95% CI [0.0196, 0.0235], *N* = 3*,* 608), GPT-3-davinci 0.0270 (95% CI [0.0235, 0.0305], *N* = 1*,* 349), ChatGPT 0.0186 (95% CI [0.0170, 0.0202], *N* = 3*,* 581), Cohere 0.0222 (95% CI [0.0205, 0.0239], *N* = 4*,* 494), LLaMA-7B 0.0230 (95% CI [0.0206, 0.0253], *N* = 2*,* 545). In general, the AIGC generated by each investigated LLM exhibits a certain degree of racial bias on toxicity at the sentence level. Among them, ChatGPT has the lowest racial bias on toxicity. It attains 0.0186 in this aspect, which indicates that, on average, the maximal absolute difference between the average toxicity score of sentences pertaining to a population group (i.e., White, Black, or Asian) in a news article generated by ChatGPT and that score in its counterpart collected from The New York Times or Reuters is 0.0186.

Figure [A.2](#_bookmark2)a reveals the magnitude of toxicity difference between each investigated LLM and the benchmark human-writing across all three racial groups. Next, we zoom in and examine the bias of each LLM against the Black race. In this context, we define a news article generated by an LLM as exhibiting Black prejudice with respect to toxicity if the average toxicity score of sentences related to the Black race in that article is higher than the average toxicity score of sentences associated with the Black race in its counterpart obtained from The New York Times or Reuters. Here, a higher toxicity score indicates a greater degree of rudeness, disrespect, and profanity. Figure [A.2](#_bookmark2)b reports the proportion of Black prejudice news articles with respect to toxicity generated by each LLM: Grover (41.08%, *N* = 869), GPT-2 (56.50%, *N* = 962), GPT-3-curie (39.04%, *N* = 978), GPT-3-davinci (39.53%, *N* = 1*,* 119), ChatGPT (32.91%, *N* = 1*,* 110), Cohere (45.43%, *N* = 1*,* 435), LLaMA-7B (34.61%, *N* = 732). For example, Grover’s performance of 41.08% shows that, for a news article obtained from the New York Times or Reuters that contains sentences associated with the Black race, there is a probability of 0.4108 that its corresponding news article generated by Grover exhibits stronger toxicity towards the Black race than the original article. Figure [A.2](#_bookmark2)C further reports the increase of toxicity score in Black prejudice news articles generated by each LLM: Grover 0.0492 (95% CI [0.0382, 0.0603], *N* = 357), GPT-2 0.0957 (95% CI [0.0812, 0.1103], *N* = 543), GPT-3-curie 0.0266 (95% CI [0.0196, 0.0337], *N* = 382), GPT-3-davinci 0.0290 (95% CI [0.0222, 0.0359], *N* = 442), ChatGPT 0.0208 (95% CI [0.0138, 0.0277], *N* = 365), Cohere 0.0239 (95% CI [0.0189, 0.0289], *N* = 652), LLaMA-7B 0.0257 (95% CI [0.0177, 0.0337], *N* = 253). Taking Grover as an example, on average, the average toxicity score of sentences related to the Black race in a Black prejudice news article generated by Grover is increased by 0.0492, compared to its counterpart collected from The New York Times or Reuters. Among the examined LLMs, ChatGPT generates the smallest percentage of Black prejudice news articles and demonstrates the slightest elevation in toxicity scores towards the Black race in these articles.

**Figure A.2. Racial Bias on Toxicity at Sentence Level. a** An LLM’s racial bias on toxicity and its 95% confidence interval (error bar), measured using Equation [6](#_bookmark1). For example, Grover’s performance of 0.0609 in this aspect, shows that, on average, the maximal absolute difference between the average toxicity score of sentences pertaining to a population group (i.e., White, Black, or Asian) in a news article generated by Grover and that score in its counterpart collected from The New York Times or Reuters is 0.0609. **b** Percentage of Black prejudice news articles with respect to toxicity generated by an LLM. We define a news article generated by an LLM as exhibiting Black prejudice with respect to toxicity if the average toxicity score of sentences related to the Black race in that article is higher than the average toxicity score of sentences associated with the Black race in its counterpart obtained from The New York Times or Reuters. **c** Increase of toxicity score in Black prejudice news articles generated by an LLM and its 95% confidence interval (error bar). Taking Grover as an example, on average, the average toxicity score of sentences related to the Black race in a Black prejudice news article generated by Grover is increased by 0.0492, compared to its counterpart collected from The New York Times or Reuters.

# B Topic Examples

Tables [B.1](#_bookmark4) and [B.2](#_bookmark5) present example topics associated with population groups pertaining to gender and racial biases, respectively. For each topic, we also report its top 15 most relevant words to illustrate the semantic content captured by the topic. We followed the suggestion of not removing stop words when training topic models[^1^](#_bookmark6), and identified content-bearing words of each topic using the relevance score as defined in LDAvis, a popular package for visualizing topic models[^2^](#_bookmark7). Topic 4 in Table [B.1](#_bookmark4) is associated with the female population group, and it is a mixed theme on art and family. Topic 51 in this table, on the other hand, is associated with the male population group, and it is about politics and famous male politicians. Topic 3 in Table [B.2](#_bookmark5) is associated with the White population group and is about the international conflict between Russia and Ukraine. The association between Topic 25 in this table and the Black population group is not surprising given that this topic is about racism and culture diversity. Topic 171 is associated with the Asian population group and it is about Asian politics.

**Table B.1.** Example Topics Associated with Population Groups Pertaining to Gender Bias

Topic 4

Association

female

Topic 51 male

Top 15 Most Relevant Words

she, her, art, artist, painting, gallery, exhibition, ms, husband, herself, daughter, painter, exhibit, mother, curator

trump, desantis, election, fox, donald, presidential, republican, candidate, campaign, indictment, governor, voter, dominion, florida, mueller

**Table B.2.** Example Topics Associated with Population Groups Pertaining to Racial Bias

Topic 3

Association

White

Topic 25 Black

Topic 171 Asian

Top 15 Most Relevant Words

russia, ukraine, russian, putin, moscow, nato, sanction, vladimir, kremlin, crimea, ukrainian, belarus, kiev, invasion, soviet black, racism, racial, diversity, culture, society, color, lorayne, disabled, racist, inclusive, zehme, cult, representation, inca china, chinese, beijing, macron, china’s, li, hong, kong, wang, communist, leyen, jinpe, shanghai, xinhua, tianjin

# References

- - 1. Schofield, A., Magnusson, M. & Mimno, D. Pulling Out the Stops: Rethinking Stopword Removal for Topic Models. In *Proceedings of the 15th Conference of the European Chapter of the Association for Computational Linguistics: Volume 2,* *Short Papers*, 432–436 (Association for Computational Linguistics, Valencia, Spain, 2017).
    2. Sievert, C. & Shirley, K. E. LDAvis: A method for visualizing and interpreting topics. In *Proceedings of the Workshop on Interactive Language Learning, Visualization, and Interfaces*, 63–70 (Baltimore, Maryland, USA, 2014).
